# Research on Electromagnetic Radiation Mechanism during Detonation of Energetic Material

**DOI:** 10.3390/s22072765

**Published:** 2022-04-03

**Authors:** Yuanbo Cui, Deren Kong, Jian Jiang, Shang Gao

**Affiliations:** School of Mechanical Engineering, Nanjing University of Science and Technology, Nanjing 210094, China; cyb6226@njust.edu.cn (Y.C.); jiangj@njust.edu.cn (J.J.); shang.gao@njust.edu.cn (S.G.)

**Keywords:** energetic material, plasma, detonation, electromagnetism, high temperature

## Abstract

In the process of deflagration of energetic materials, strong electromagnetic radiation is generated, which causes the surrounding electronic equipment to fail to work normally. To solve this problem, it is necessary to clarify the mechanism of electromagnetic radiation generated by energetic materials. The mechanism of plasma changed by the deflagration of energetic materials is an important topic in the aerospace and geophysics fields. The academic community holds two main viewpoints on the mechanism of electromagnetic radiation generated by energetic materials: one is that the solid material is squeezed and deformed during the deflagration of energetic materials, and the charges of different polarities rub in space to form effective electric dipoles, which eventually generate electromagnetic radiation. Another view is that the deflagration of energetic materials causes the temperature of the medium to rise sharply, and bremsstrahlung is formed during the compression and diffusion of the high-temperature wave front, resulting in the generation of electromagnetic radiation. This paper, based on theoretical analysis and experimental data, holds the view that electromagnetic radiation is generated by the high-temperature thermal effect. It studies the relationship between temperature and electromagnetic radiation and obtains quantitative analysis conclusions.

## 1. Introduction

**Figure 1:**
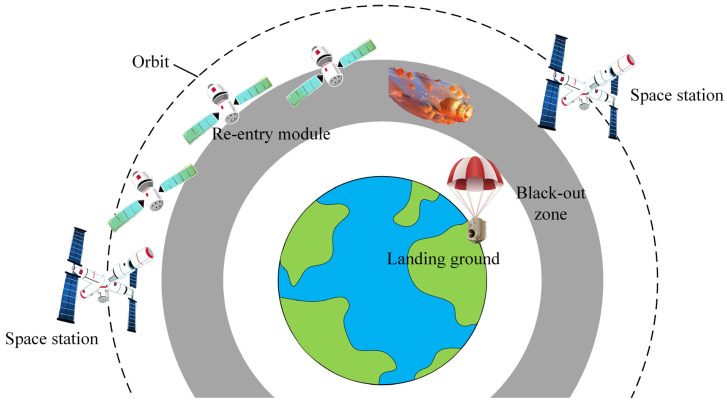
The return process of Shenzhou XII spacecraft.

China launched the Shenzhou XII spacecraft from the Jiuquan Satellite Launch Center on 17 June 2021, its seventh manned spaceflight, sending three astronauts to the core module of the Tianhe Space Station. After completing a three-month work task, the Shenzhou XII spacecraft returned to Earth and successfully landed at the Dongfeng Landing Site on 17 September 2021. Figure 1 shows that when spacecraft return to the earth, due to the excessive speed, the surface of the spacecraft and the surrounding gas friction generate substantial heat, forming a high-temperature plasma gas layer on the surface of the spacecraft, which blocks electromagnetic waves and causes the spacecraft to lose contact with the ground.

There are very few studies on the mechanism of plasma changed by the deflagration of energetic materials. Boronin conducted earlier in-depth research on the mechanism of electromagnetic radiation generated by energetic materials. He took condensed explosives as the object of study and proposed that at the moment when the metal shell of the explosive is deformed and broken, on a large number of experimental data, the difference in the outflow velocity of the gaseous products and the solid products produces charges of different polarities. As the dipole moment continues to increase, the electromagnetic intensity continues to increase, and electromagnetic interference occurs. After the pulse extrema passes, the charge in the detonation conducting region begins to relax until the fragments fall into hot air with high conductivity, the detonation conductive area loses charge, and the dipole moment and electric field strength decrease until the detonation electromagnetic effect disappears. Boronin expounded for the first time the physical mechanism and complete process of the electromagnetic radiation generated by the detonation, which is of great significance to subsequent related research [1,2]. To explore the mechanism of electromagnetic radiation generated by energetic materials, Chen Shengyu conducted a series of experiments, including an experiment measuring electromagnetic radiation generated by the thermal bridge wire when the shielded cable supplies power to the electric detonator, and an experiment measuring electromagnetic radiation generated by energetic materials. Chen finally deduced the correlation between the electromagnetic radiation generated by energetic materials and the dynamic parameters of detonation. Chen believes that the main dynamic parameter affecting the electromagnetic radiation of energetic materials is velocity. Through theoretical derivation, the dimensions and variables involved in the measurement of electromagnetic radiation produced by energetic materials are obtained, including the mass of energetic materials, detonation velocity, dielectric constant, and viscosity parameters [3,4,5]. Dai Qing [6] holds the view that the huge energy generated at the moment of the detonation of energetic materials causes the gas temperature to rise rapidly to 3500 K, resulting in the ionization of the gas to form plasma. The free electrons in the plasma pass around the positive ions and lose their energy under the action of the electric field, resulting in bremsstrahlung. In addition, the free electrons in the plasma are captured by the ion electric field through various differentiation processes into electromagnetic waves radiated when the electrons are bound, that is, compound radiation. The spectra of bremsstrahlung and composite radiation are superimposed to form a continuous spectrum covering a certain frequency range, which becomes the source of electromagnetic radiation [7]. Chen Hong believes that in the detonation process of condensed explosives, the detonation wave, detonation products, and air shock wave form an aerodynamic field and radiated energy to the space around the air. The wave front air is instantly heated to 10,000~12,000 K, resulting in the formation of a nonequilibrium and incomplete low-temperature plasma region near the explosion, in which the charged components generate a quasi-stationary electric and magnetic field, the field strength of which is limited by the explosion heat, shock wave velocity, explosive product ion concentration, and other factors [8].

Regarding the mechanism of electromagnetic radiation generated by energetic materials, two mainstream views have been formed in the scientific community, but the existing experimental data are few, which causes the research to remain at the theoretical stage, and there is no basis to support the relevant views [9]. In this paper, by measuring the electromagnetic radiation in the detonation process of high-energy energetic materials and recording the temperature data with an infrared camera, combined with theoretical analysis, the plasma state transformation caused by high temperature is confirmed, which is of great significance to research in the field of high-temperature plasma. The research results of this paper can help to evaluate the interference degree of electromagnetic radiation generated by energetic materials on electronic equipment, provide help for electronic equipment in resisting electromagnetic interference, and ultimately improve the stability of electronic systems.

## 2. Theoretical Analysis

Through in-depth research on the plasma state generated by the air explosion shock wave, it is found that the explosion is similar to the high-speed shock problem, and it is a dynamic process with fast energy deposition with the density of explosives and the material at the front of the shock wave being higher. Multiple sets of ionization models were improved to evaluate the ionization state of the generated plasma, and the ionization model was combined with the finite volume method based on AUSM+-up to simulate the plasma generated in the air shock wave. The atoms collide with each other, and the energy generated by the motion is transferred to the electrons in the atoms. When the transfer energy reaches a threshold value, the electrons are separated from the atoms. The formation reaction process of plasma can be expressed by Equation (1). *A*^+^ represents the positive ions generated by ionization, *e*^−^ represents the generated electrons, and the collision of ions and electrons leads to the reverse reaction of ionization. When the two reaction rates are similar, the ionization process reaches an equilibrium state, and the collision process between particle *a* and particle *b* can be expressed as Equation (2).

(1) $$A⇌A^{+}+e^{-}$$

(2) $$\left\{ \begin{array}{l} Z_{ab}=n_{a}n_{b}\sigma_{ab}u_{ab}\\ u_{ab}=\sqrt{\frac{8kT_{a}}{\pi m_{a}}+\frac{8kT_{b}}{\pi m_{b}}} \end{array}\right.$$

In Equation (2), *u_ab_* is the relative thermal velocity and obeys the Maxwell distribution. The average velocity of the Maxwell distribution is used here because it is suitable for describing the collision between particles, and *σ_ab_* is the collision cross section between particles *a* and *b* and can be expressed as *σ_ab_* = *πd*^2^. *d* is the average particle size in the hard sphere model, and *n_a_* and *n_b_* are the number densities of particle *a* and particle *b*, respectively, so the collision frequency between each particle can be expressed by Equation (3). *Z_ea_* is the collision frequency between electrons and atoms, *Z_ea+_* is the collision frequency between electrons and ions, and *Z_aa_* is the collision frequency between atoms. In the calculation of *σ_C_*, the diameter of the heavy particle in the hard sphere model is selected as *d*. The differences between atoms and ions other than electric charge are ignored here, and the collision frequency of heavy particles and electrons is replaced by *Z_ea_* due to the weak ionization of the blast shock wave. Therefore, the three equations in Equation (3) are sufficient to describe the reaction and heat conduction processes in the plasma generated by the blast shock wave [10,11,12,13,14].

(3) $$\left\{ \begin{array}{l} Z_{ea}=n_{e}n_{a}\sigma_{C}\sqrt{\frac{8kT}{\pi m_{a}}+\frac{8kT_{e}}{\pi m_{e}}}\\ Z_{ea+}=n_{e}n_{a}+\sigma_{C}\sqrt{\frac{8kT}{\pi m_{a}}+\frac{8kT_{e}}{\pi m_{e}}}\\ Z_{aa}=\frac{1}{2}n_{a}{}^{2}2\sigma_{C}\sqrt{2\frac{8kT}{\pi m_{a}}} \end{array}\right.$$

The relationship between the shock wave and ionization state and the effect of ground reflection on the ionization state are studied by two typical simulations: in the initial stage of detonation, the central part of the detonation product is ionized. As the detonation shock wave propagates, the degree of ionization in the central part decreases, the ionization zone moves as the shock front propagates through the air, and the ionization zone follows the shock wave. As the shock propagates, the ionization zone separates from the shock front, and the degree of ionization drops by 2 orders of magnitude from the start of the simulation to the end of the simulation. The study found that the air shock wave pressure and the ionization degree of the ionization simulation numerical center area at different times during the explosion process decreased much faster than the ionization degree near the shock front. From this, we can know the degree of ionization of the shock-induced plasma caused by the propagation of the shock wave. play an important role, which also means that the reflection of the shock wave can significantly affect the plasma generation. When the shock wave is reflected on the ground, the ionization degree in the reflection zone of the shock wave increases rapidly, which is 2~3 orders of magnitude higher than that in the free expansion zone. Therefore, compared with the original plasma behind the free-propagating shock wave, the plasma caused by this rapid growth will generate additional electromagnetic pulses. This phenomenon confirms that the interaction between the shock waves has a significant effect on the plasma ionization degree, which can be considered to be the principle of electromagnetic pulse generation of energetic materials [15,16,17,18].

**Figure 2:**
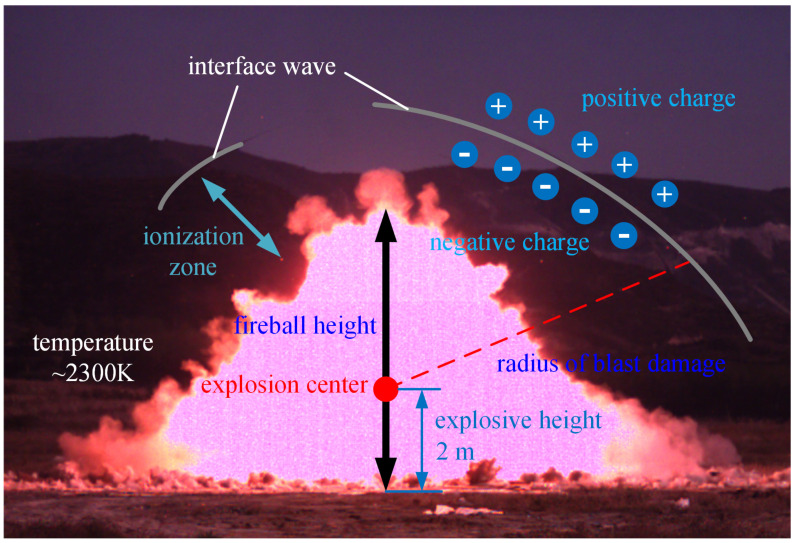
Schematic diagram of plasma changes caused by detonation at high temperature.

There are two mechanisms for generating electromagnetic radiation during the detonation of energetic materials: one is that the deformation of the working medium is affected by the piezoelectric effect, friction, gas ionization, etc., resulting in a large number of free charges, and the movement of free charges generates electromagnetic radiation; the other is that the temperature rises to generate thermal stress. The thermal stress is caused by the particles inside the working medium squeezing and repelling each other, thereby generating moving charged particles and finally electromagnetic radiation. A schematic diagram of plasma changes caused by detonation at high temperature is shown in Figure 2.

As shown in Figure 2, it can be seen from Equation (1) that for a reaction system in an equilibrium state, the free energy of the system should satisfy the relationship shown in Equation (4), where *F* is the free energy, *N* represents the number of various particles in the system, *N_e_* represents the neutral atom, *N*_+_ means positive ion, and *N*_−_ means electron. The relationship between the free energy of the system and the energy distribution function *Z* is shown in Equation (5), where *k* is the Boltzmann constant (*k* = 1.38065 × 10^−23^ J/K), and *T* is the temperature [19,20,21].

(4) $$\frac{\partial F}{\partial N_{e}}-\frac{\partial F}{\partial N_{+}}-\frac{\partial F}{\partial N_{-}}=0$$

(5) F=−kTlnZ

The partition function of Equation (5) can be expressed as a function of the configuration integral *Q_N_*, where *Q_N_* is a quantity related to the system potential energy as *U_P_* (*r*_1_, *r*_2_, …, *r_N_*), the gas density in the detonation ionization process of energetic materials is very high, and potential energy must be considered. It can be assumed that the potential energy is uniformly distributed in the gas, the electron is an ideal gas, the interaction between the electron and the ion is replaced by the potential energy of the ion, and the ion and the atom have the same potential energy. The potential energy can be expressed as Equation (6), where *E_p_* represents a particle possessing potential energy.

(6) $$\left\{ \begin{array}{l} U_{p0}(r_{1},r_{2},\ldots ,r_{N0})=N_{e}E_{p}\\ U_{p1}(r_{1},r_{2},\ldots ,r_{N1})=N_{+}(E_{p}+I_{+}) \end{array}\right.$$

Therefore the configuration function can be expressed as Equation (7), where *V* represents the volume of the ionized system.

(7) $$\left\{ \begin{array}{l} Z=\frac{Q}{\lambda^{3N}N!}\\ Q_{N}=\exp(-\frac{NE_{P}}{kT})\int \cdots \int d_{r1}d_{r2}\cdots d_{rN}=\exp(-\frac{NE_{P}}{kT})V_{N}=\exp(-\frac{NE_{P}}{kT})V^{N} \end{array}\right.$$

The overall free energy of an ionized system can be described as the sum of the neutral atomic free energy, ionic free energy, and electron free energy, as shown in Equation (8), where *m_e_* is the electron mass and *m_a_* is the gas atomic mass.

(8) $$\begin{array}{l} F=kTN\ln\frac{\lambda^{3}{}_{T}}{V}+kTN_{e}\ln N_{e}-kTN+kTN_{+}\ln N_{+}\\ +E_{P}N+I_{+}N_{+}+kTN_{-}\ln\frac{\lambda^{3}{}_{Te}}{V}+kTN_{-}\ln N_{-}-kTN_{-} \end{array}$$

Finally, by substituting Equation (8) into Equation (4), the final relationship of ionization is obtained in Equation (9). The particle number density relationship of each component in the thermal equilibrium ionization of a real gas is obtained, as shown in Equation (9). Under the assumption that the potential energy is uniformly distributed in each particle, the potential energy has no effect on the ionization process, and only has a significant effect on the temperature under the same internal energy. This conclusion theoretically demonstrates that the detonation thermal effect of energetic materials produces electromagnetic radiation [22,23,24,25]. Parameters such as *m_e_*, *k*, and *h* in Equation (9) are all constants, and Equation (9) can be simplified to Equation (10), which shows that the plasma particle motion activity *v*(*n*_−_, *n*_+_, *n_e_*) is positively correlated with the plasma temperature. When the temperature of plasma becomes higher, the particles in the plasma move faster, and the friction effect of the plasma is more obvious, so stronger electromagnetic radiation is finally produced.

(9) $${\left(\frac{m_{e}kT}{2\pi h^{2}}\right)}^{\frac{3}{2}}\exp(-\frac{I_{+}}{kT})=\frac{n_{-}n_{+}}{n_{e}}$$

(10) $$T\propto v(n_{-},n_{+},n_{e})$$

## 3. Experimental Results

**Figure 3:**
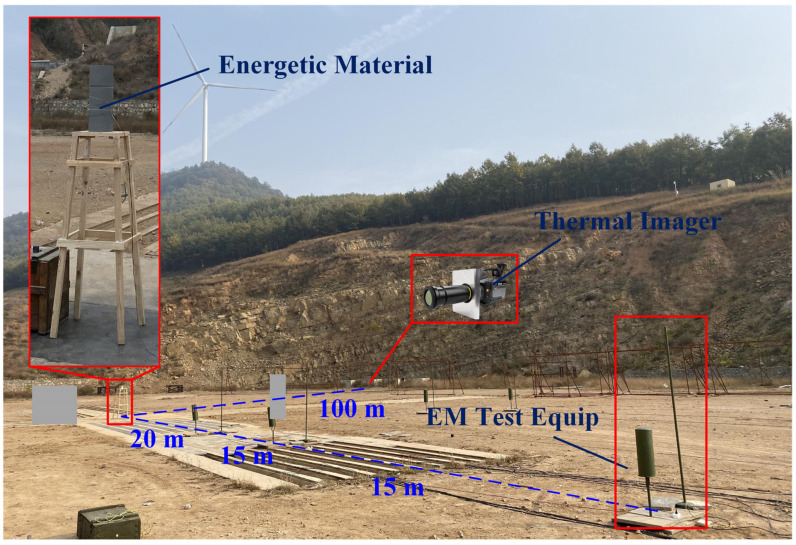
Experimental field and equipment.

An electromagnetic radiation measurement device based on an ultra-wideband antenna and a shortwave antenna was designed to measure the characteristics of electromagnetic radiation generated by energetic materials. The experimental field and experimental equipment are shown in Figure 3. The test point consisted of a shortwave antenna, an ultra-wideband antenna, and a signal coupler. The shortwave antenna had a sampling frequency of 1 MHz–30 MHz, and the output impendence was 50 Ω. The ultra-wideband antenna had a sampling frequency of 30 MHz–1.5 GHz, and the output impendence was 50 Ω. The signal coupler could combine two electromagnetic signals of different frequencies and amplify the signal at the same time with a range of amplification factors of 10 dB–30 dB. The experimental site was selected on flat land surrounded by mountains. The weather on the day of the experiment was sunny, the atmospheric temperature was 11 °C, and the relative humidity was 18%. Four common high-energy explosives were selected as the energetic materials: Hexogen (RDX), Trinitrotoluene (TNT), Pentaerythritol tetranitrate (PETN), and Octogen (HMX). The mass of the energetic materials used in the experiment was 600 kg. To improve the accuracy of the test results, six test points were placed in the test field, and each of the three test points formed a column, at 20 m, 25 m, and 50 m away from the energetic material. In a bunker 100 m from the energetic material, thermal imaging instruments were placed to measure the temperature of the plasma during the detonation process. The thermal imaging instrument had a temperature measurement range of −40 to 2500 K and frame rate of 250 fps, and it had a thermal sensitivity of less than 15 mK with an accuracy of 1% of the reading value [26].

**Figure 4:**
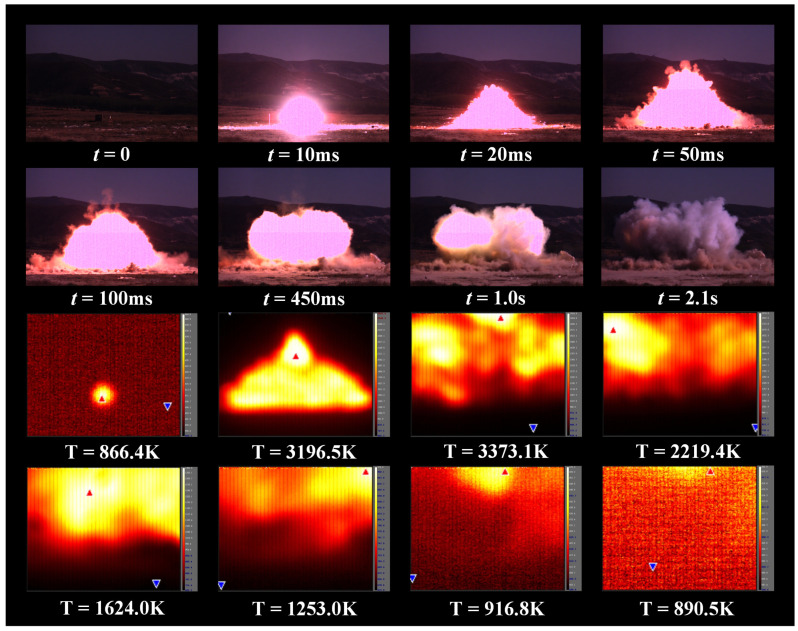
Temperature measurement for the detonation process of RDX.

**Figure 5:**
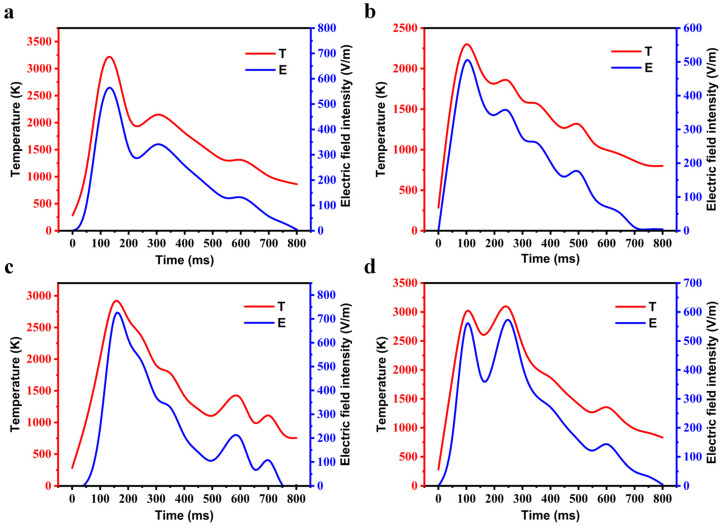
Variation diagram of temperature and electromagnetic intensity during detonation of energetic materials: (**a**) RDX; (**b**) TNT; (**c**) PETN; (**d**) HMX.

Measurement experiments were carried out on the detonation process of four energetic materials: Hexogen (RDX), Trinitrotoluene (TNT), Pentaerythritol tetranitrate (PETN), and Octogen (HMX), and the test items included the intensity of electromagnetic radiation and the surface temperature of the plasma. The results of the RDX temperature measurement experiment are shown in Figure 4, from which it can be obtained that the duration of the detonation process was approximately 2.1 s, and the fireball gradually disappeared after 1.0 s. RDX detonation can produce temperatures up to 3373.1 K. After the RDX was detonated, the plasma temperature increased sharply from 866.4 K to 3196.5 K and started to decrease after the plasma temperature reached the highest value of 3373.1 K. Compared with the temperature increase rate, its decreasing rate was relatively slow, and the temperature during the whole detonation process was always kept above 800 K. The variation diagram of temperature and electromagnetic intensity during detonation of energetic materials is shown in Figure 5, in which subfigures (a–d) represent the temperature change and the electromagnetic intensity change of the four kinds of energetic materials (RDX, TNT, PETN, HMX) during detonation, respectively.

After the RDX detonation, the plasma temperature reached a maximum value of 3373.1 K at 150 ms, and then dropped rapidly to 1846.5 K at 200 ms. The temperature increased slightly and then slowly decreased until the temperature still reached 860.5 K at 800 ms. The intensity of electromagnetic radiation generated by RDX detonation reached a maximum value of 563.5 V/m at 150 ms. After the detonation of TNT, the plasma temperature increased rapidly, reaching a maximum value of 2301.3 K at 100 ms, while the electromagnetic radiation intensity reached a maximum value of 504.7 V/m, and then the temperature curve and the electromagnetic intensity curve decreased in waves, and the plasma temperature remained at 800 K until 800 ms. After the PETN detonation, the plasma temperature rose to 2900.8 K at 150 ms, and the electromagnetic radiation intensity reached 703.6 V/m, and then the two parameters began to decrease, and the downward trend remained until 500 ms. The plasma temperature fluctuated from 500 ms to 800 ms, and the intensity of electromagnetic radiation also fluctuated. After the HMX detonation, two obvious peaks appeared in the plasma temperature. The temperature was 3010.3 K at 100 ms and 3074.2 K at 250 ms, and the plasma temperature decreased steadily after 300 ms. The electromagnetic radiation intensity also exhibited two obvious peaks. The intensity was 556.9 V/m at 100 ms and 572.9 V/m at 250 ms, and the electromagnetic radiation intensity decreased steadily after 300 ms. In summary, in the detonation process of energetic materials, there was a very obvious correlation between the changes in plasma intensity and temperature, so it can be considered that the electromagnetic radiation of energetic materials is caused by the changes in plasma temperature.

## 4. Conclusions

The wide application of energetic materials in the aerospace field has made aviation technology more advanced. However, some problems have also occurred in the process: for instance, the spacecraft passes through the atmosphere on its way back to Earth and causes intense combustion, which generates strong electromagnetic radiation, posing a threat to the spacecraft’s electronic equipment. The mechanism of plasma change caused by the deflagration of energetic materials has always been an important topic explored by physicists, and two mainstream views are as follows: 1. High-speed irregular motion of energetic material particles forms electric dipoles, which generate electromagnetic radiation. 2. The detonation of energetic materials causes the surrounding temperature to rise sharply, and the high-temperature thermal effect causes electromagnetic radiation. To study the mechanism of electromagnetic radiation produced by energetic materials, a measurement system composed of electromagnetic measuring equipment and temperature measuring equipment was designed to measure the parameters of the detonation process of energetic materials (RDX, TNT, PETN, HMX). The electromagnetic radiation intensity and plasma temperature data of the detonation process of the four energetic materials were obtained in the experiment. By comparing the changing trends of the electromagnetic radiation intensity and the plasma temperature during the detonation process, it was found that the two were highly consistent. Therefore, this article supports the view that the high-temperature thermal effect forms bremsstrahlung that produces electromagnetic radiation. This paper fills the gap in the experimental data for the measurement of high-equivalent energetic materials and uses the experimental data to verify the view that high temperature generates electromagnetic radiation, which is reliable and valuable.

## Data Availability

Not applicable.
